# Employing active learning in the optimization of culture medium for mammalian cells

**DOI:** 10.1038/s41540-023-00284-7

**Published:** 2023-05-30

**Authors:** Takamasa Hashizume, Yuki Ozawa, Bei-Wen Ying

**Affiliations:** School of Life and Environmental Sciences, University of Tsukuba, 1-1-1 Tennodai, Tsukuba, 305-8572 Ibaraki Japan

**Keywords:** Systems analysis, Cell biology, Computer modelling

## Abstract

Medium optimization is a crucial step during cell culture for biopharmaceutics and regenerative medicine; however, this step remains challenging, as both media and cells are highly complex systems. Here, we addressed this issue by employing active learning. Specifically, we introduced machine learning to cell culture experiments to optimize culture medium. The cell line HeLa-S3 and the gradient-boosting decision tree algorithm were used to find optimized media as pilot studies. To acquire the training data, cell culture was performed in a large variety of medium combinations. The cellular NAD(P)H abundance, represented as A450, was used to indicate the goodness of culture media. In active learning, regular and time-saving modes were developed using culture data at 168 h and 96 h, respectively. Both modes successfully fine-tuned 29 components to generate a medium for improved cell culture. Intriguingly, the two modes provided different predictions for the concentrations of vitamins and amino acids, and a significant decrease was commonly predicted for fetal bovine serum (FBS) compared to the commercial medium. In addition, active learning-assisted medium optimization significantly increased the cellular concentration of NAD(P)H, an active chemical with a constant abundance in living cells. Our study demonstrated the efficiency and practicality of active learning for medium optimization and provided valuable information for employing machine learning technology in cell biology experiments.

## Introduction

It is increasingly important to develop approaches that can efficiently optimize culture medium, as developing culture medium for mammalian cells is essential in the medical and biopharmaceutical fields^1^. In addition to developing cell lines^2–4^, culture media have been intensively studied to improve the performance of developed cell lines^5^. The composition of the culture medium, e.g., carbon sources, amino acids, fatty acids, vitamins, trace elements, and salts^6^, usually must be optimized for cell growth and production^7–9^. However, it is challenging to optimize medium because the influence of components in medium on cellular metabolism is complex^10^. The conventional one-factor-at-time (OFAT) method fine-tunes the medium components individually^11^, but this approach is time-consuming and inefficient. The statistical method design of experiments (DOE) is efficient when less than 10 medium components must be adjusted^12^. Response surface methodology (RSM)^13,14^ uses the quadratic polynomial approximation, which may be too simple to represent the comprehensive interaction between the medium and cell^15^. Machine learning (ML) has been used to overcome these limitations^16^.

ML has generally been used to develop predictive models based on the relationships among features of a given dataset. Its workflow commonly involves processing input data, training the underlying model, and predicting new data^17^. In recent years, ML has been increasingly applied in biological studies^18^, particularly for analyzing large or highly complex datasets^16^. As a representative example, the cell culture medium is a highly structured dataset that commonly comprises hundreds of components as variable features. Optimizing medium components is essential for performing cell cultures, which are widely used in the food industry, pharmaceutical development, medical therapy, etc^12^.. ML has been tested for medium development^19,20^ and has shown higher performance than that of DOE and RSM, the commonly used approaches^15,21^.

The efficiency of medium optimization should depend on the prediction accuracy of the ML model. Active learning has been proposed as a method to improve prediction accuracy with a small dataset by allowing ML models to select data for training^22,23^. This approach has been practical in drug discovery^24–26^ and has successfully been used to optimize the buffer composition for protein biosynthesis in a cell-free system^27^. However, applying active learning to optimize media for mammalian cell culture remains under investigation. For instance, in the past 20 years, 37% of studies on the development of medium for CHO cells considered only one additive, 37% used OFAT, 15% used DOE, and none used machine learning or active learning^28^. Researchers that optimized the medium for other cell lines used ML algorithms without active learning^19,29^. Whether active learning can be applied to optimize the culture medium of mammalian cells is an intriguing topic.

In the present study, active learning combining explanatory ML with experimental validation was used to optimize the medium composition to improve cell culture^28,30^. Although all medium components influence cell growth and production, when considering the contributions of medium components to cell culture, researchers have mainly focused on amino acids, which are critical for cell viability, growth, and bioproduction^31,32^. The other components remain largely under investigation. To address this issue, the present study employed a gradient-boosting decision tree (GBDT), a white-box ML algorithm, instead of the widely used black-box ML algorithms, e.g., neural networks (NNs). Owing to its high interpretability, GBDT has been broadly used in the fields of medicine^33–35^, pharmacy^36^, ecology^37^, and so on. Our previous study used GBDT to explore the contribution of medium components to bacterial growth and successfully observed the chemical dependence of different components as the survival strategy^38^. By applying GBDT in active learning to optimize the medium for mammalian cells, we can identify the contribution of individual medium components to cell culture.

## Results

### Experimental design for acquiring the training data

**Fig. 1:**
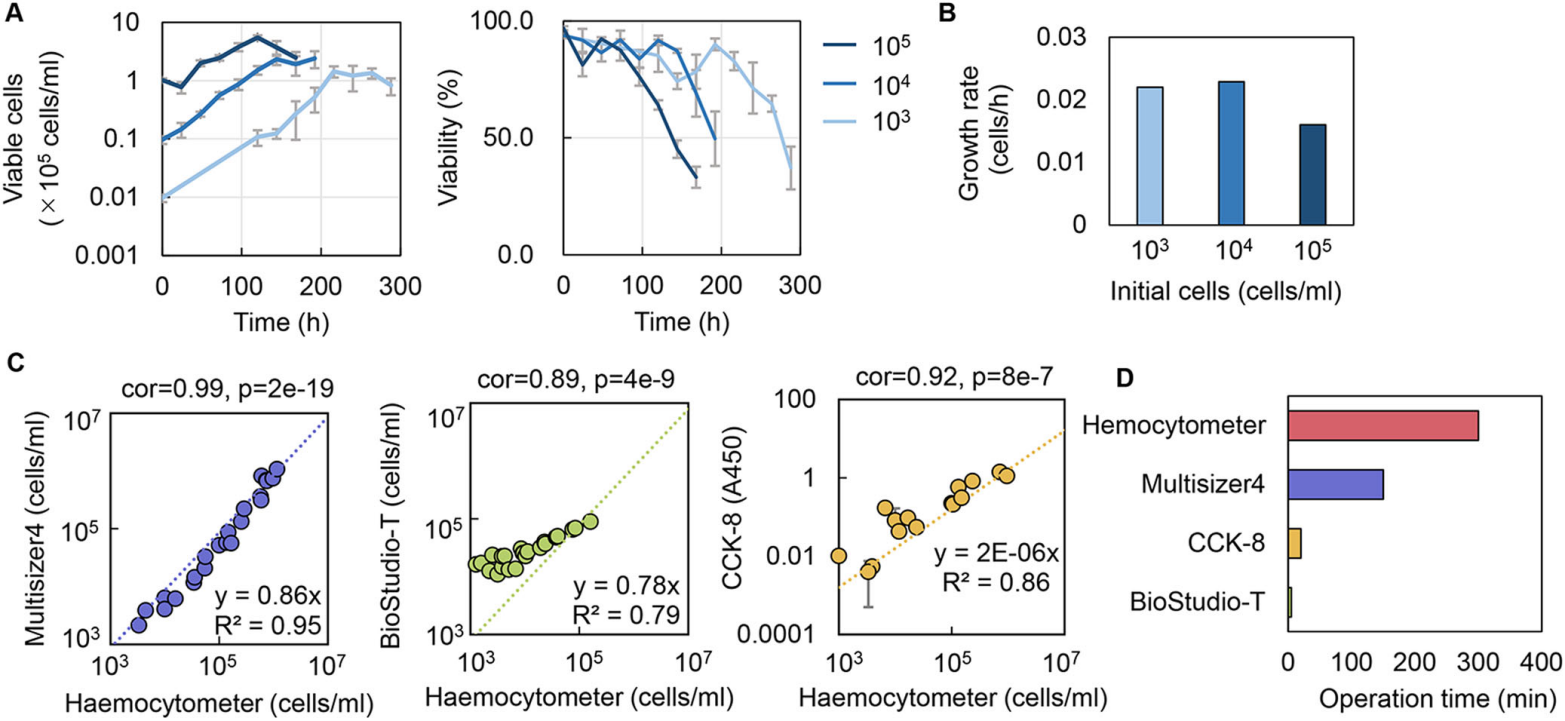
Determination of experimental approaches for data collection. **A** Temporal changes in cell culture and cell viability at various initial cell concentrations. The color gradation indicates the variation in initial cell concentration. Standard errors (s.d.) of biological replicates (*N* = 4) are indicated. **B** Cell growth rates evaluated by exponential approximation. The color gradation indicates the variation in the initial cell concentration. **C** Various methods for cell counting. Spearman correlation coefficients and *p* values are indicated. The dotted lines represent the linear regression shown with the equations and *R*^2^. Errors (s.d.) of biological replicates (*N* = 2) are indicated. **D** Time required to evaluate the cell culture in a 96-well microplate. The time for the experimental measurement was evaluated, i.e., after the 96-well microplate was removed from the incubator for measurement to when the measurement of cell abundance (A450, counting, etc.) of a microplate was completed.

**Fig. 2:**
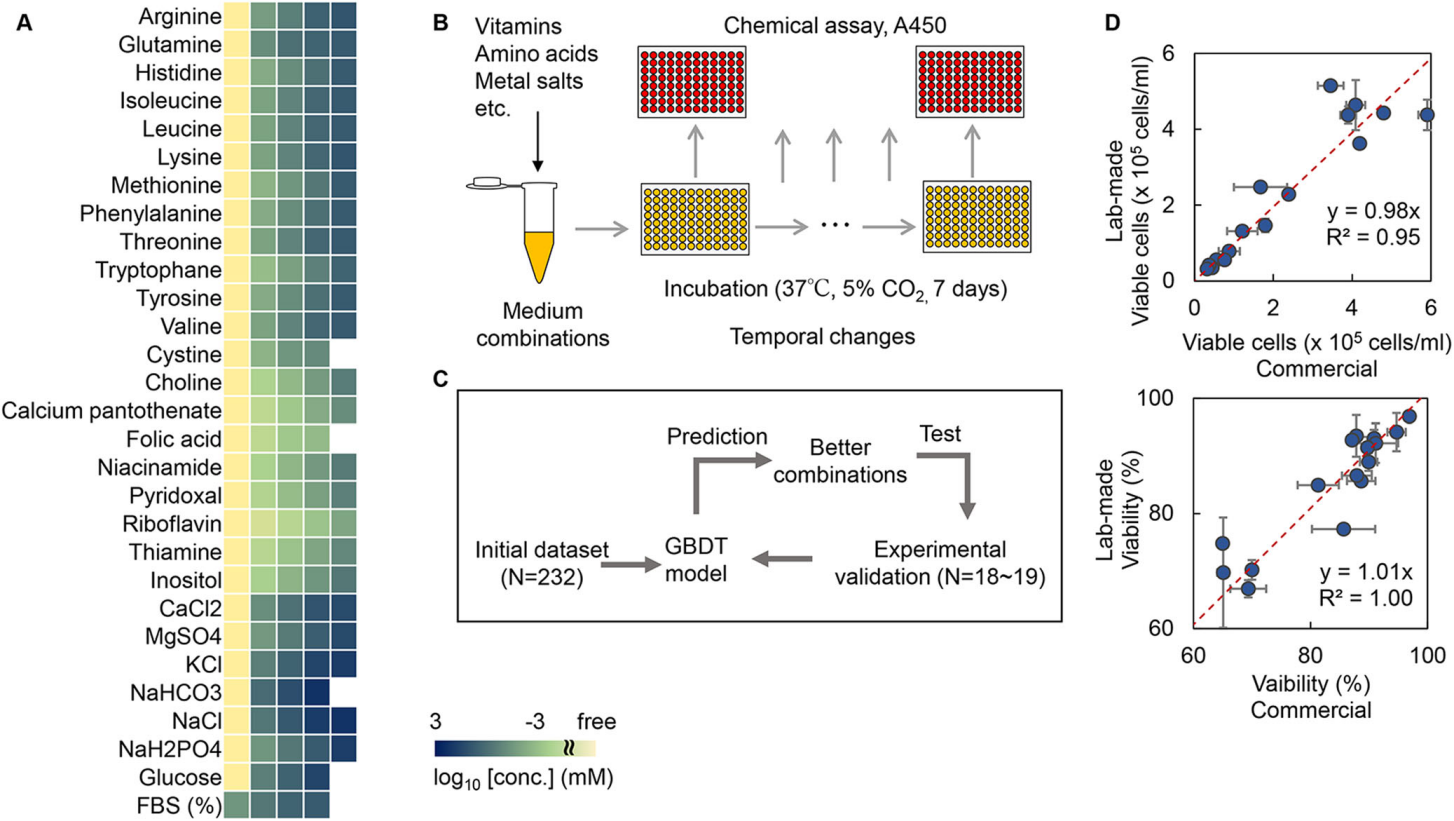
Experimental data acquisition and active learning. **A** Medium components and their concentrations used in the initial dataset for active learning. Every four to five concentration gradients, varying from zero- to 10–100-fold of the concentrations in EMEM, were set for each chemical component. Only one component was changed in each medium combination, and a total of 232 combinations were finally acquired and used as the initial dataset. Color gradation indicates the concentration gradients shown on a logarithmic scale. **B** Schematic drawing of the experimental procedure for experimental data acquisition. **C** Flowchart of active learning for medium optimization. **D** Comparison of commercially purchased and laboratory-made EMEM media. The upper and bottom panels show the number of viable cells and the viability, respectively. HeLa cells cultured in 24-well plates for various times (24–336 h) are shown. Standard errors (s.d.) of biological replicates (*N* = 2) are indicated. The red lines indicate the slope of 1.

The cell line HeLa-S3 was used in the study, as it could grow in suspension mode and was thus easy to evaluate. The initial cell concentration was determined at 10^4^ cells/ml, as lower and higher concentrations led to an extended culturing time (Fig. 1A) and reduced growth rate (Fig. 1B). Quantitative evaluation of the cell culture was achieved through testing multiple methods, i.e., counting the cell particles (Multisizer), cell imaging analysis (BioStudio-T), chemical reaction assay (CCK-8), and cell stain and counting (Haemocytometer). When comparing these methods to the most reliable and commonly used method using Haemocytometer, the methods using Multisizer and CCK-8 were preferred according to the correlation coefficients (Fig. 1C). Considering the time needed for operation and the measurement range of cell concentration (Fig. 1D), the chemical reaction assay using CCK-8 upon the cellular NAD(P)H abundance was selected, in which the cell concentration was evaluated through the absorbance at 450 nm (A450). This method was efficient and convenient for acquiring an extensive dataset for ML, as it could be performed in a high-throughput manner. In addition, the medium components subjected to optimization were determined according to the composition of the commonly used Eagle’s minimum essential medium (EMEM), which comprised approximately 31 components. Except for phenol red and penicillin‒streptomycin, 29 components were used to prepare the medium combinations for active learning (Fig. 2A). The concentration gradients of these components were varied on a logarithmic scale to acquire a broad data variation and were not biased from biological measurements or experimental experience. Through the wide range of chemical concentrations, ML could search the medium combinations that were never tested by conventional medium optimization. Finally, cell culture in 232 medium combinations was performed, and the temporal changes in cell culture were measured at 24- or 48-h intervals (Fig. 2B). Biological replicates (*N* = 3–4) were conducted for each sampling point, resulting in thousands of A450 records. Note that any potential manual (personal) bias in preparing the medium combinations irrelevantly affected the cell culture, as the commercially purchased and the lab-made media (EMEM) showed approximately equivalent cell concentration and viability (Fig. 2D).

### Regular and time-saving modes of active learning for medium optimization

**Fig. 3:**
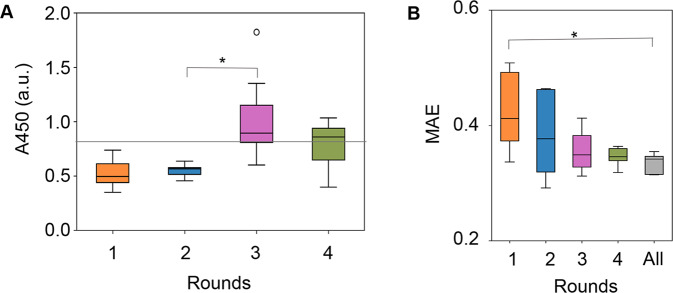
Regular mode of active learning for medium optimization. **A** Boxplots of A450 of the cell culture at 168 h. Every 18–19 medium combinations predicted by the GBDT model using the culture data at 168 h are tested. Mean values for biological replicates (N = 3) are shown. The gray horizontal line indicates the cell culture with EMEM. An arbitrary unit is shown as a.u. **B** Boxplots of the prediction accuracy of the GBDT model built in each round. Mean absolute error (MAE), representing the average of the absolute difference between predicted and measured A450 values, was calculated by fivefold nested cross-validation. “All” indicates the accuracy evaluated using the entire dataset from the initial to Round 4. Asterisks indicate statistical significance by Mann‒Whitney’s *U*-test (*p* < 0.05). The boxplots show the median (center line), interquartile range (bounds of box), the range of typical data values (whiskers), and outliers (circle).

Active learning was performed to search for the medium combination that improved cell culture. As a regular mode of active learning, A450 of the cell culture at 168 h was used for the training because it was roughly the time point at which saturated cell concentration was reached (Fig. 1A). The GBDT model was used to predict the medium combinations leading to a better cell culture, i.e., higher A450, and the learning loop was started with the dataset comprising 232 medium combinations (Fig. 2C). Every 18–19 predicted combinations were subjected to experimental validation. The experimental results were added to the training data. The procedures for model building, medium prediction, experimental validation, and training (Fig. 2C) were repeated four times (Fig. 3A). As a result, both the A450 values of the cell culture and the accuracy of the GBDT models were elevated. The cell culture was significantly improved in round 3 and remained comparable after round 4 (Fig. 3A). We assumed that either the methodology or the cell culture reached its limitation. Note that for the following round of ML, the tested media were not better than EMEM, i.e., 74% and 58% showed better cell culture than EMEM in rounds 3 and 4 (Supplementary Fig. 1A). The best media were always better than EMEM after round 3 (Supplementary Fig. 1B). The prediction accuracy improved gradually as the number of rounds increased (Fig. 3B and Supplementary Fig. 2A). Thus, active learning successfully fine-tuned the medium combination associated with increasing ML accuracy.

**Fig. 4:**
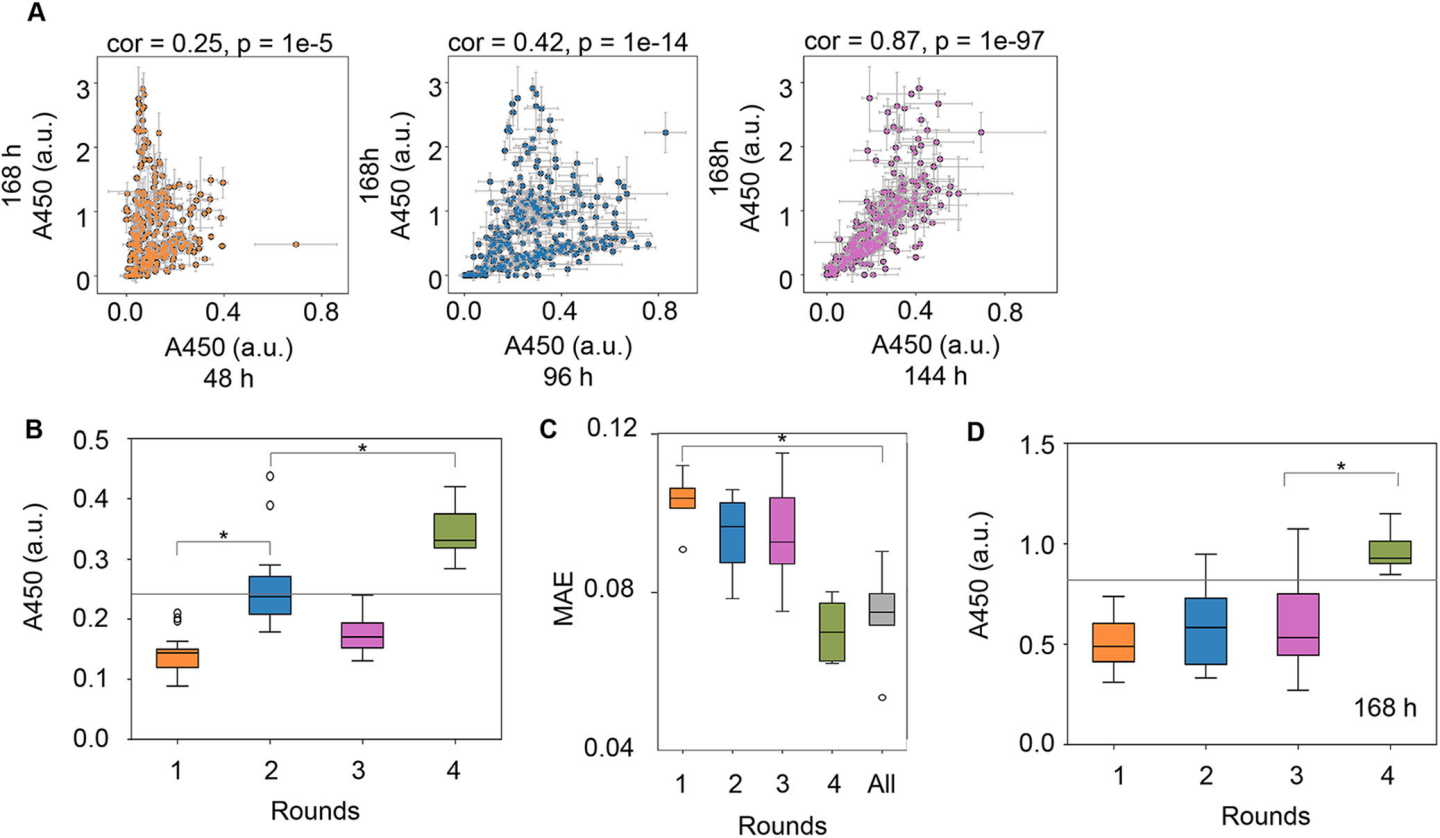
Active learning for medium optimization in time-saving mode. **A** Relationship of the cell culture at various time points. Correlations of the cell culture at 168 h to those at 44, 96, and 144 h are shown in orange, blue, and purple, respectively. The cell culture was evaluated at A450. Spearman correlation coefficients and *p* values are shown. Standard errors (s.d.) of biological replicates (*N* = 3) are indicated. **B** Boxplot of A450 of the cell culture at 96 h. Every 18–19 medium combinations predicted by the GBDT model using the culture data at 96 h were tested. **C** Boxplot of the prediction accuracy of the GBDT model built in each round. MAE was calculated by fivefold nested cross-validation. “All” indicates the accuracy evaluated using the entire dataset from the initial to Round 4. **D** Boxplot of A450 of the cell culture at 168 h. Every 18–19 medium combinations predicted by the GBDT model using the culture data at 96 h were tested. Mean values for biological replicates (*N* = 3) are shown. The gray horizontal lines indicate the cell culture at 96 or 168 h with EMEM. Asterisks indicate statistical significance by the Mann‒Whitney *U*-test (*p* < 0.05). The boxplots show the median (center line), interquartile range (bounds of box), the range of typical data values (whiskers), and outliers (circles). An arbitrary unit is shown as a.u.

Subsequently, whether the active learning loop could be shortened was tested. As the data acquisition step (168 h) was time-consuming, it was investigated whether A450 of the cell culture at an earlier time could be used to predict the cell culture at 168 h. Significant correlations in A450 were detected between the earlier time points (48, 96, and 144 h) and the endpoint at 168 h (Fig. 4A). Although A450 at 144 h showed the best correlation to that at 168 h, the time point of 96 h was selected because the time was effectively shortened. Consequently, A450 of the cell culture at 96 h was employed as a time-saving mode for active learning. The 232 medium combinations associated with the A450 values of the cell culture at 96 h were employed as the initial dataset for active learning. Both the A450 of the cell culture at 96 h and the prediction accuracy were improved (Fig. 4B, C and Supplementary Fig. 2B), as observed in the regular mode. Intriguingly, A450 of the cell culture at 168 h was significantly increased (Fig. 4D), even though ML used the culture data acquired at 96 h. The medium combinations for improved cell culture were successfully achieved when the culture data was used earlier than necessary, which shortened the hundreds of hours needed for medium optimization in the present case (Supplementary Fig. 3). Note that no significant improvement was achieved even if the additional round was performed with the time-saving mode (Supplementary Fig. 4). In addition, the correlation of A450 at 96 h to that at 168 h was higher in active learning (Supplementary Fig. 5). The changing ratio of A450 between 96 and 168 h followed the high ratio, despite the initial bimodal distribution of high and low changing ratios observed for A450 between 96 and 168 h (Supplementary Fig. 5). This result indicated that the initial data distribution slightly affected the ML. These results demonstrated that the time-saving mode was practical for ML-assisted medium optimization to improve cell culture.

### Contribution and composition of optimized medium components

**Fig. 5:**
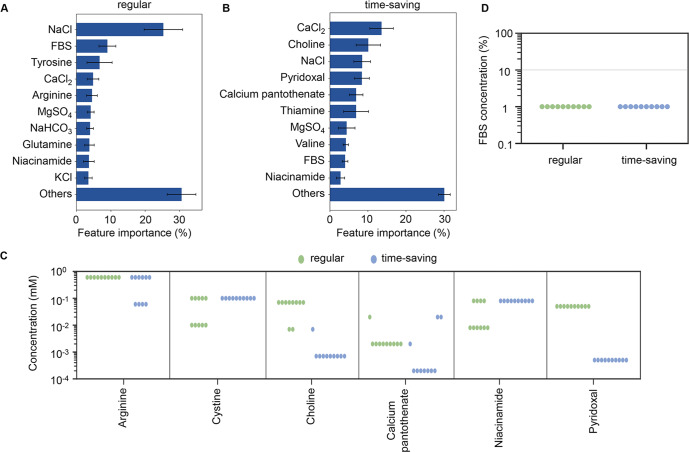
Contribution and composition of the optimized media. **A** Relative contribution of medium components to cell culture at 168 h (regular mode). The top ten components and the sum of the remaining 19 components, i.e., Others, are indicated. Fivefold cross-validation of GBDT in the regular mode was applied. Standard deviations of five replicates are indicated. **B** Relative contribution of medium components to cell culture at 96 h (the time-saving mode). The top ten components and the sum of the remaining 19 components, i.e., Others, are indicated. Fivefold cross-validation of GBDT in the time-saving mode was applied. Standard deviations of five replicates are indicated. **C** Chemical component concentrations in the optimized media predicted by the regular and time-saving modes. The six chemical components, in which the concentrations were significantly different between the two modes, are shown. The green and blue circles represent the media optimized by the regular and time-saving modes. **D** Abundance of FBS in the optimized media. The concentrations of FBS in the optimized media predicted by the regular and time-saving modes are indicated as green and blue circles, respectively. The gray horizontal line indicates the serum concentration in the commercial EMEM medium.

The contribution of medium components to cell culture was estimated using GBDT. All datasets acquired through active learning, i.e., 302 and 403 medium combinations in the regular and time-saving modes, respectively, were used (Supplementary Figs. 6 and 7). The importance of each component in the two modes was estimated. The top ten components that mainly contributed to cell culture partially overlapped (Fig. 5A, B). This result indicated that both the regular and time-saving modes fine-tuned similar components, e.g., metal salts and FBS, for improved cell culture. This finding partially explained why the medium optimization at 96 h improved cell culture at 168 h. Intriguingly, NaCl and CaCl_2_, but not FBS, were the primary components that determined the cell culture in the regular and time-saving modes, respectively. Although FBS usually contains calcium ions^39^ and 1–3 mM CaCl_2_ is generally supplied in cell culture^6^, adjusting the concentration of calcium ions is essential for cell growth because either excess or deficient calcium ions induce cell apoptosis^40–43^. In addition, high osmotic pressure, which is caused by the high concentration of NaCl in the medium, arrests cell growth^44^. The results indicated that the cellular permeability, which is regulated by NaCl and CaCl_2_, might present the highest priority in cell culture rather than the growth factors provided by FBS.

The compositions of the best ten media predicted with the regular mode, which optimized A450 at 168 h, and the time-saving mode, which optimized A450 at 96 h, were compared. The concentrations of most components in the media predicted by the two modes were comparable to those in EMEM (Supplementary Fig. 8). Six components showed significant differentiation in concentrations of either amino acids or vitamins between the two modes (Fig. 5C). This indicates that the regular and time-saving modes specifically optimized amino acids and vitamins, respectively. Interestingly, the concentration of FBS in all 20 media predicted by the two modes was one order of magnitude lower than that in the commonly used EMEM^6^, although the compositions of other components were varied in these media (Fig. 5D). FBS contains a variety of factors essential for cell growth, such as trace elements (vitamins and minerals), hormones, free radical scavengers and mitogenic growth factors^45^, which are absent in the basal media^46^. Generally, 10–20% FBS in the media is suitable for cell culture, as reported in enterocytes^47^, stem cells^48^, and hybridomas^49^. This reduced amount of FBS should be a preferable trend, considering the risk of contamination, the cost, and the batch-to-batch variability in cell production^50,51^. Arginine in the regular mode and choline, pantothenic acid, niacinamide, and pyridoxal in the time-saving mode were significantly different between the regular and time-saving modes (Fig. 5D). This indicates that in regular mode, the amino acids necessary for cell survival in the late phase of culture were adjusted, while in time-saving mode, the vitamins that protect cells from oxidative stress in the early phase of culture were adjusted. In conclusion, different chemical components, with vitamins in the early phase and amino acids in the late phase, may affect cell culture.

### Comparison of the optimized media to the original medium

**Fig. 6:**
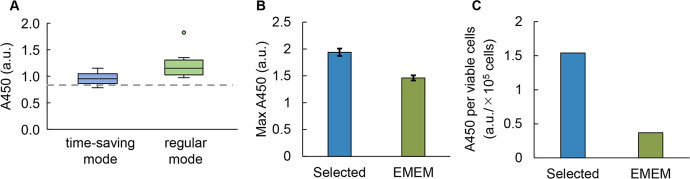
Performance of the ML-optimized media. **A** Comparison of the optimized media predicted with the regular and time-saving modes. The top ten media, which showed the highest A450 absorbance of cell culture, are shown. The dashed line indicates the cell culture in EMEM. The boxplots show the median (center line), interquartile range (bounds of box), the range of typical data values (whiskers), and outliers (circle). **B** Comparison of A450 between the selected and EMEM media. The selected medium is the medium that achieved the highest A450 in round 3 of the regular mode. The concentrations of choline and pyridoxal were tenfold higher and those of folic acid and FBS were tenfold lower in the selected medium compared to EMEM. Thiamine was absent in the selected medium. Standard errors (s.d.) of biological replicates (*N* = 2) are indicated. **C** Comparison of cellular A450 between the selected and EMEM media. The ratio between the mean A450 and the mean number of viable cells is shown. An arbitrary unit is shown as a.u.

The optimized media showed higher A450 than that of the original medium EMEM, regardless of the regular and time-saving modes. The best ten medium combinations (i.e., those that showed the highest A450) predicted with the regular and time-saving modes were prepared to experimentally verify the cell culture at 168 h. The predicted media showed better performance, i.e., higher A450, than that of the original medium EMEM (Fig. 6A). Thus, both modes resulted in successful medium optimization, although a better performance was obtained by the media predicted with the regular mode than those predicted with the time-saving mode.

In addition, a direct comparison of the optimized medium to the original medium was performed. The best of the 20 predicted media was selected for comparison with the commercially purchased EMEM. The cell culture was performed in a 1-mL volume as a scale-up compared to the culture volume used in active learning. The selected medium showed higher A450 (Fig. 6B), as well as increased A450 per cell (Fig. 6C), which failed to improve the viable cell concentration (Supplementary Fig. 9). Active learning of medium optimization likely increased the cellular abundance of NAD(P)H more significantly than the total abundance of NAD(P)H. This finding made us reconsider whether the commonly used chemical assay for cell culture solely provides the cell concentration. The methodology used for experimental data collection plays a crucial role in the final output of the improved parameters.

## Discussion

Cell culture is a time-consuming process in medium development. A much longer time is usually necessary for mammalian cell culture than biochemical reactions using cell extracts and bacterial cultures. Medium composition is often quickly optimized by statistically reducing the experimental test conditions^52^. An alternative approach to acquire the experimental dataset for ML-assisted medium optimization is shortening the data acquisition time for quick optimization. The present study was a challenging trial to reduce the experimental time. The information on the early phase culture successfully predicted the output of the late phase culture (Fig. 4). This indicates that time-saving active learning is practical in accelerating medium development. Nevertheless, further verification is necessary to ensure that the current approach is practical in optimizing culture media for biomedical applications and biopharmaceutical production, which is highly essential^53^.

In the present study, active learning of medium optimization successfully increased cellular NAD(P)H abundance but not the concentration of cells, which was unexpected. NAD(P)H abundance is proportional to the number of viable cells^54^ based on the assumption that NAD(P)H abundance per viable cell is constant. However, our results showed that the cellular NAD(P)H abundance could be increased by optimizing the medium (Fig. 6). We assume that the optimized media was beneficial for metabolism and increased NAD(P)H abundance in cells. The upper limit of cellular NAD(P)H abundance might be why active learning failed to increase A450 after round 4 (Fig. 3). In an alternative view, the cellular capacity of NAD(P)H could be addressed by active learning. This finding provided us with an ideal approach to search for the biological limitation of living cells, e.g., the highest growth rate, through active learning. Additionally, the results demonstrated that the ML-combined medium optimization was highly sensitive to the biological parameter, e.g., cellular activity or amount, that was chosen as the variable in ML.

High priorities of vitamins and amino acids were predicted by the two modes when determining the cell culture. As the time-saving and regular modes used the culture data at 168 h and 96 h, diverse components predicted by the two modes might reflect the cellular physiological differentiation in the early and late phases. In the time-saving mode, five of the ten determinant components were vitamins (Fig. 5), which are closely related to the cellular NAD(P)H abundance mediated by the stress response component reactive oxygen species (ROS). The choline pathway synthesizes serine and glycine, which are necessary for antioxidant glutathione synthesis^55^ and were absent in the media. Pyridoxal inhibits the formation of superoxide radicals^56^. Pantothenic acid prevents ROS-induced apoptosis^57^. Niacinamide is known as the amide of niacin, a precursor of NAD^+^ and NADP^+^^58^. The high priority of these vitamins indicated that they play a crucial role in deciding the early phase of cell culture. In the regular mode, the determinants were tyrosine, arginine, and glutamine. The abundance of amino acids was reported to be related to cell viability^59^, as an excess amount was reported to accumulate toxic metabolites^60^. Metabolisms of the three amino acids were found to produce ammonia, 4-hydroxyphenylpyruvate, and dimethylarginine^61^, which are toxic to cells^62,63^ and cause growth inhibition^64^ or cell apoptosis^65,66^. The high priority of these amino acids strongly suggested that an adequate amount was essential to maintain balanced metabolism for cell viability in the late phase of cell culture. Taken together, we assumed that the amino acids promoted cell growth in the late phase and that the vitamins protected the cells from oxidative stress in the early phase. Further transcriptome and metabolome analyses are essential to clarify the differentiated requirement of amino acids and vitamins in various cell culture phases.

The study demonstrated that active learning was effective in medium optimization and that using time-saving mode with data from the early culture phase was practical. Nevertheless, whether active learning can be used for media development for other cell lines or substance production remains to be examined. As a quick reference, once the media optimized for the HeLa cell were tested with another cell line CHO, an increase in cellular NAD(P)H abundance was observed (Supplementary Fig. 10). Although the optimized media were useful in culturing different cells, media optimization using the target cell line is preferred to obtain the best performance of cell culture. Additional training data are needed for constructing the ML models. Living cells fluctuate even under stable conditions^67,68^, and their working principles remain largely unclear. ML-assisted medium optimization is assumed to be limited within the cell line and the purpose of culture for growth or production. Employing additional parameters related to cellular physiology, e.g., cell size and gene expression, may lead to a better and more efficient ML model for medium optimization.

In addition to biological restrictions, additional issues should be considered to improve the accuracy of ML with fewer experiments. First, uncertainty sampling is commonly employed to improve ML models in active learning^23^. In the present study, uncertainty sampling was absent in the datasets, as only the medium combinations for better cell culture were selected for experimental validation in active learning. Therefore, additional experimental data of worse cell culture, such as uncertainty sampling, for training was necessary to improve the accuracy of ML models to further develop the culture medium. Second, the overfitting or underfitting issues, which occurred once the ML models were too complex or too simple^17^, were thought to influence the performance of ML. The success in ML-assisted medium optimization revealed that the ML model built in the present study was neither overfitting nor underfitting. Here, the ML model was built using existing Python libraries with adjusted hyperparameters. Although most ML algorithms were developed outside the biological field and used for nonbiological purposes^16^, our results demonstrated that it was possible to construct ML models accurately processing biological data using existing libraries without requiring highly professional computational techniques. The simple application of ML to medium optimization was achieved due to a considerable effort to fine-tune the experimental operations and obtain biological data that fit ML technology. The present study provided an example of how to apply ML to biological studies. Although many issues remain, the successful trial provides valuable knowledge to further develop ML-assisted medium optimization for biomedical applications and biopharmaceutical production.

## Materials and methods

### Cell line and culture

The commonly used mammalian cell line, HeLa-S3, was obtained from the RIKEN Cell Bank (Tsukuba, Japan). Cell culture was performed at 37 °C in a CO_2_ incubator (E-50, As One) supplied with 5% CO_2_^69^. Cells were cultured in multiwell plates (Iwaki) with a culture volume of 0.5 or 1 mL in 48- or 24-well microplates, respectively. Multiple wells were used for biological replicates. The cultured cells were detached with PBS (-) (Wako) supplemented with trypsin-EDTA (Sigma). The cells were collected by centrifugation and resuspended in cryopreservation solution (Wako) with trypan blue (Wako). The cells were counted in a haemocytometer (DHC-N01, Nano Entek) with a microscope (ECLIPSE Ts2, Nikon)^70^. The number of viable cells was evaluated accordingly.

### Preparation of cell stocks

Cell stocks for repeated cultures were prepared to reduce experimental errors^71^. The cells stored in liquid nitrogen were thawed at 37 °C and suspended in 4 ml of Eagle’s minimum essential medium (EMEM, Wako) for medium exchange. Subsequently, the cells were collected and suspended in 10 mL of EMEM supplemented with 1% penicillin‒streptomycin solution (Wako) and 10% FBS (Japan Bio Serum). The cells were cultured in 10 cm dishes (Violamo) for two days at 37 °C in a CO_2_ incubator and were counted in a haemocytometer, as described above. The cell culture was divided into 1 mL, dispensed into 1.2 ml cryotubes (Biosigma), frozen at −80 °C for 24 h, and finally stored in liquid nitrogen for future use. As a result, dozens of identical cell stocks were prepared.

### Preparation of medium combinations

The medium combinations were prepared with 31 commercially available compounds, in which choline chloride and pyridoxal hydrochloride were from Tokyo Chemical Industry, FBS was from Japan Bio Serum, and the remaining compounds were from Wako. The abundance of penicillin‒streptomycin and phenol red were maintained at 1% and 0.03 mM, respectively. The concentrations of FBS were changed in the range of 0.1–10%. The remaining 28 components were changed zero- to 10–100-fold of their concentrations in EMEM. In brief, four to five different concentrations were used for each component, and the changes were roughly on a logarithmic scale, because a broad range of concentration gradients benefited the ML-assisted optimization^38^. The medium combinations were prepared by mixing the stock solutions of the chemical compounds, which were individually prepared in advance, with highly pure water (Direct-Q UV, Merck). The stock solutions were sterilized by sterile syringe filters (Merck) with hydrophilic PVDF membranes of 0.22 µm pore size, dispensed in a small volume and stored at −20 °C for future use. Note that all medium combinations were prepared immediately before use, and the stock solutions were used once. All medium combinations tested in the present study were summarized in Supplementary Data 1 and Supplementary Data 2 for the regular and time-saving modes, respectively.

### Cell counting according to single-particle analysis

A particle size analyzer (Multisizer 4, Beckman Coulter) was used to count the number of cells. The cell culture in a 48-well microplate (Iwaki) was suspended in 10 ml of ISOTON II (Beckman Coulter). Every 100–500 µl of the suspended solutions were flowed for particle analysis, according to the manufacturer’s instructions. Particles within the range from 8 to 12 µm in diameter were gated as the cells. The mean value of the biological replicates was calculated as the cell concentration.

### Cell counting by imaging analysis

The cells cultured in a 48-well microplate (Iwaki) were imaged with BioStudio-T (Nikon) according to the manufacturer’s instructions. The image of the cell culture was analyzed with the software NIS-Elements (Nikon), and the number of cells was evaluated automatically.

### Temporal changes in cell culture evaluated by chemical assay

The cell culture was performed in 200 µl with 96-well microplates (Iwaki), and the culture conditions were described above. As explained elsewhere^72^, only the wells in the middle of the plate (60 wells) were used for cell culture to prevent evaporation. Multiple microplates of identical cell cultures were prepared for temporal evaluation of the cell culture. These plates were subjected to the chemical assay sequentially at 48, 96, 144, and 168 h. Ten microlitres of CCK-8 (Dojin) was added to the cell culture and incubated at 37 °C for one hour, according to the protocol. Finally, 20 µl of 0.1 M HCl was added to arrest the reaction, and the absorbance at 450 nm was measured with a plate reader (Epoch 2, BioTek). A450 was used as the relative cell concentration for machine learning.

### Data processing

Absorbance reads of the chemical assay were exported from the plate reader and processed with Python, in which the mean A450 of biological replicates was calculated using “mean” in the “numpy” library. The actual A450 of the cell culture was calculated by subtracting the mean A450 of the medium. The datasets obtained in the present study are summarized in Supplementary Data 1 and 2.

### Machine learning

The gradient-boosting decision tree (GBDT) algorithm was used in machine learning (ML), which was performed with Python. The “GradientBoostingRegressor” from the “ensemble” module of the “scikit-learn” library was used to construct the ML model, where the medium components and the A450 of cell culture were employed as the explanator and the objective variables, respectively. Fivefold cross-validation and grid search were performed to search for hyperparameters, which used “GridSearchCV” in the “model_selection” module of the “scikit-learn” library. The hyperparameters were searched for “learning_rate” from 0.001 to 0.5 in increments of 0.005, “max_depth” from 2 to 5 in increments of 1, and n_estimators fixed at 300. The other hyperparameters were used by default. In addition, the “feature_importances_“ attributed to the GBDT model constructed in the outer fivefold cross-validation was used to calculate the feature importance. Gini feature importance was used, which was calculated by computing the mean squared error (MSE) at each node of the decision tree and then calculating the degree to that of reduced MSE by partitioning by features^73,74^. Five replicates were performed, and the mean values were used as the final evaluation.

### Active learning for medium optimization

Active learning in either regular or time-saving mode was performed with a supercomputer, the Cygnus system (NEC LX 124Rh-4G). The A450 values obtained at 168 h and 96 h during cell culture were used as objective variables in the regular and time-saving modes, respectively. According to the ML model constructed with the initial training dataset, approximately 10 million candidate medium combinations were obtained by altering the concentrations of the medium components with four to five variations. By inputting the 10 million candidate media into the ML model, the relative cell culture, represented by A450, was predicted. The top 18 or 19 medium combinations of high A450 were selected and subjected to experimental verification. The experimental results were added to the training dataset for the following learning and prediction. Learning, prediction, and validation were performed repeatedly to achieve improved cell culture, that is, as high A450 as possible. In addition, the experimental results were used to evaluate the prediction accuracy of the ML models.

### Evaluation of the ML models

Fivefold nested cross-validation was performed to calculate the prediction accuracy of the ML models, in which the hyperparameters in the inner 5-fold cross-validation were adjusted using grid search. The five scores computed in the outer 5-fold cross-validation were used for prediction accuracy. Three metrics were employed to evaluate the prediction accuracy: mean absolute error (MAE), coefficient of determination (*R*^2^), and root mean square error (RMSE). MAE and *R*^2^ were calculated using the “mean_absolute_error” and “ r2_score “ in the “metrics” module of the “scikit-learn” library. RMSE was calculated using “mean_squared_error” in the “metrics” module of the “scikit-learn”. The square root of the MSE was calculated using “sqtr” in the “numpy” library.

### Reporting summary

Further information on research design is available in the Nature Portfolio Reporting Summary linked to this article.

## Supplementary information

Supplementary figures

Reporting summary

Sypplementary data 1

Supplementary data 2

## Data Availability

All data generated or analyzed during this study are included in this published article and its Supplementary Information files (Supplementary Data 1 and 2).

## Code availability

Code is available at https://github.com/hashizume711/medium_optimization.
