# Supplementary material for: Employing active learning in the optimization of culture medium for mammalian cells
Source: NPJ Syst Biol Appl. 2023 May 30;9:20. doi: 10.1038/s41540-023-00284-7 (PMC10229643; doi:10.1038/s41540-023-00284-7)
Supplement: Supplementary file 1 — Supplementary figures [file 41540_2023_284_MOESM1_ESM.pdf]

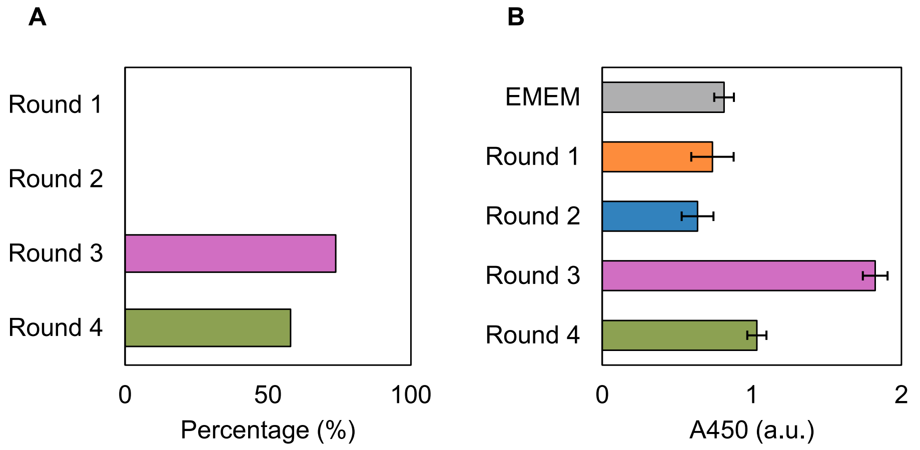

Supplementary Figure 1 Comparison of EMEM to the tested media predicted by regular mode. A. Ratio of the tested media of higher A450 than EMEM. The number of 18~19 tested media predicted by the regular mode, which achieved higher A450 than EMEM, was counted in each round. The ratio of the number to the total number was calculated. **B.** Optimized media tested in each round. The tested media that achieved the highest A450 in each round are shown with EMEM**.** Standard errors (s.d.) of biological replicates (N=3) are indicated.

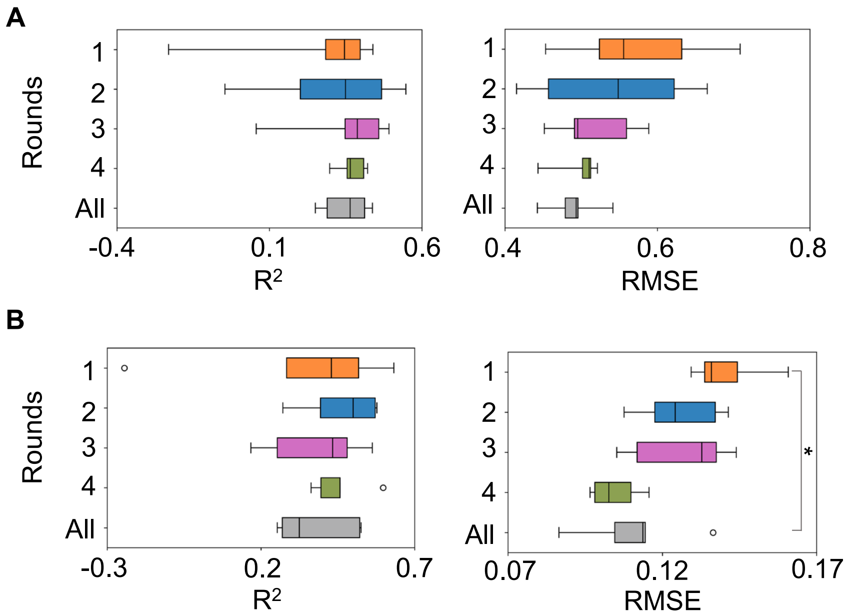

Supplementary Figure 2 Prediction accuracy of the ML models. A. Boxplots of the prediction accuracy in the regular mode. **B.** Boxplots of the prediction accuracy in the time-saving mode. The Left and right panels indicate the metrics of R^2 and RMSE, respectively. "All" indicates the accuracy evaluated using the entire dataset from the initial to Round 4. Asterisks indicate statistical significance by Mann-Whitney's U test ($p < 0.05$). The boxplots show the median (center line), interquartile range (bounds of box), the range of typical data values (whiskers), and outliers (circles).

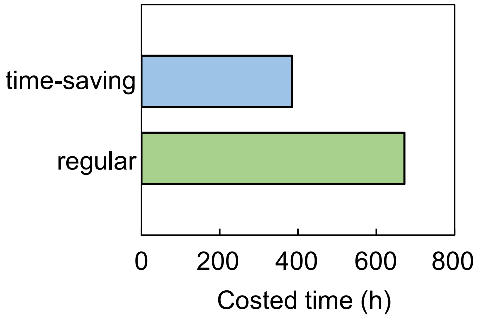

Supplementary Figure 3 Time cost for active learning. The total time cost for the four rounds of active learning was summed.

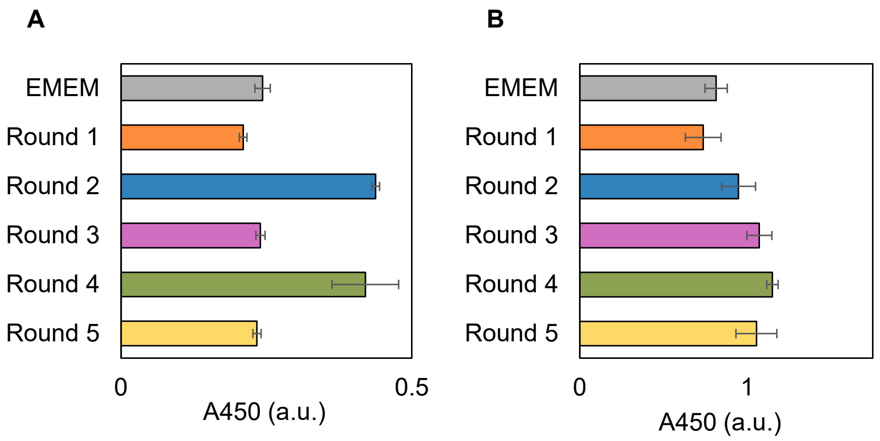

Supplementary Figure 4 Best media predicted by time-saving mode. The tested media predicted by time-saving mode in each round showing the highest A450 at 96 h **(A)** and 168 h **(B)** are shown. Standard errors (s.d.) of biological replicates (N=3) are indicated.

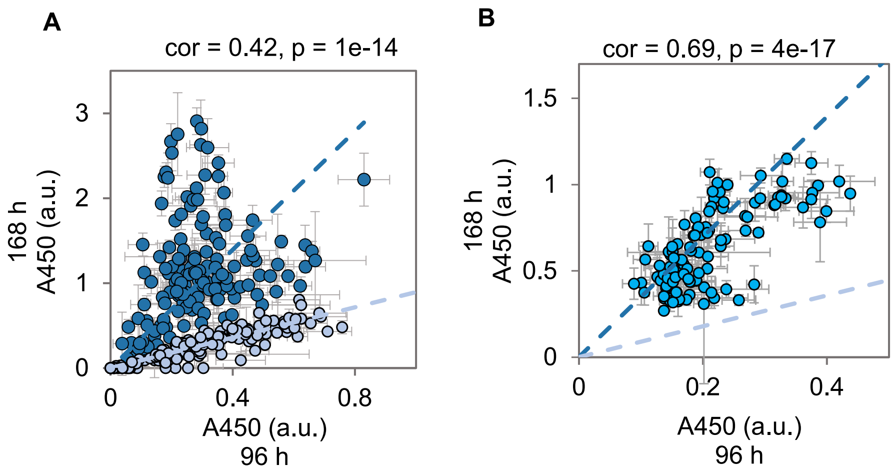

Supplementary Figure 5 Correlation of the cell culture at 168 h to those at 96 h. The A450 values acquired in the initial media (**A**) and the tested media predicted by time-saving mode from round 1 to round 4 (**B**) are shown. Broken lines indicate linear regression of the two populations of varied slopes. The high and low ratios (slopes) are highlighted in dark and light blue, respectively. Standard errors (s.d.) of biological replicates (N=3) are indicated. Spearman correlation coefficients and p-values are shown.

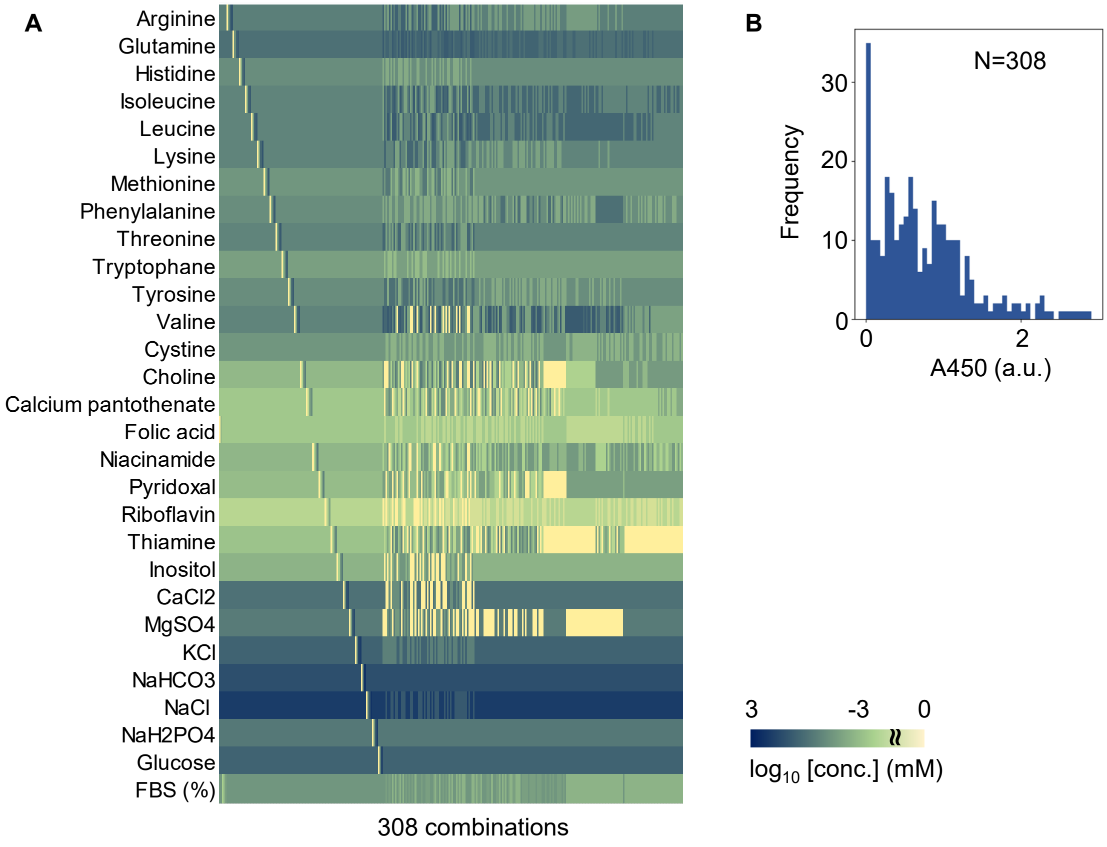

Supplementary Figure 6 Medium combinations used in the regular mode. A. Medium combinations used in active learning. A total of 308 medium combinations are shown in the heatmap. Concentrations are shown on a logarithmic scale. **B.** Distribution of A450 of the cell culture at 168 h. The number of medium combinations is indicated.

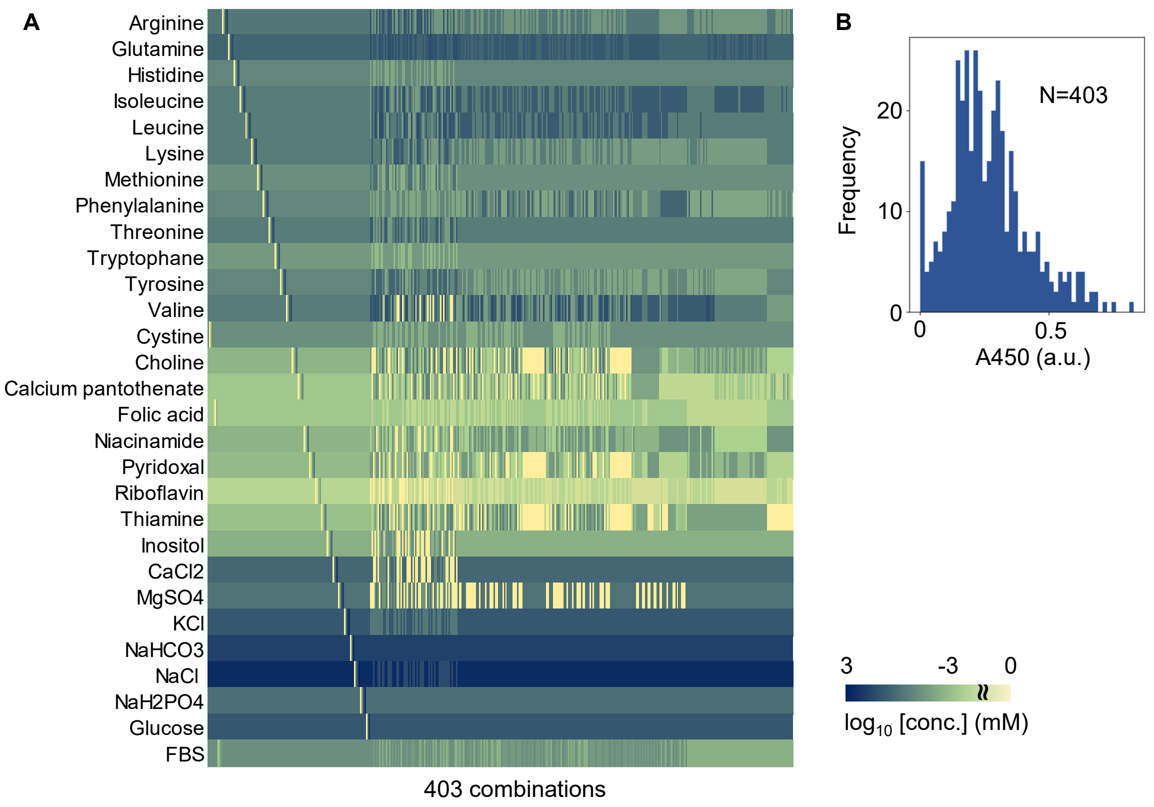

Supplementary Figure 7 Medium combinations used in the time-saving mode. A. Medium combinations used in active learning. A total of 403 medium combinations are shown in the heatmap. Concentrations are shown on a logarithmic scale. **B.** Distribution of A450 of the cell culture at 96 h. The number of medium combinations is indicated.

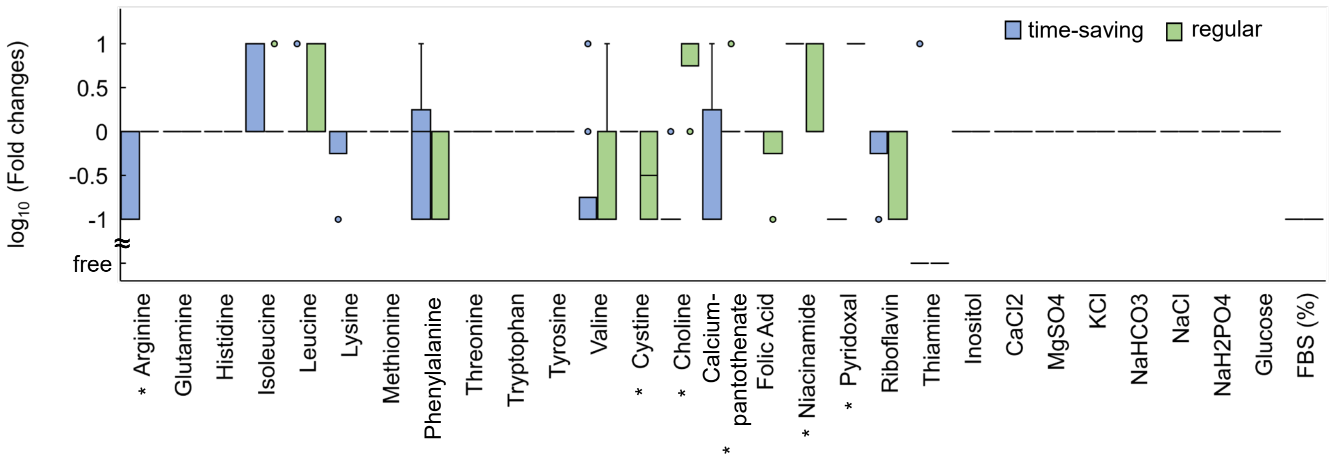

Supplementary Figure 8 Boxplot of the fold change in the concentration of a component in the optimized media versus EMEM medium. Concentrations of the 29 components in the top ten media optimized for each mode are compared to those in EMEM. Green and blue represent the media predicted with regular and time-saving modes. Fold changes in concentration are shown in the logarithmic scale. Asterisks indicate statistical significance by Mann-Whitney U-test ($p<0.05$). The boxplots show the median (center line), interquartile range (bounds of box), the range of typical data values (whiskers), and outliers (circles).

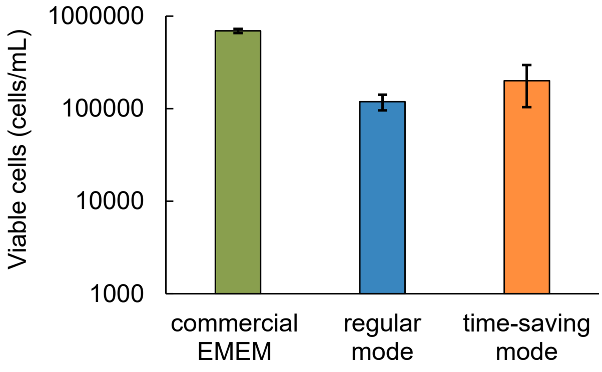

Supplementary Figure 9 Viable cell concentration. The cell concentrations at 168 h in the optimized media predicted by the regular and time-saving modes are compared to that in EMEM. Standard errors (s.d.) of biological replicates (N=2) are indicated.

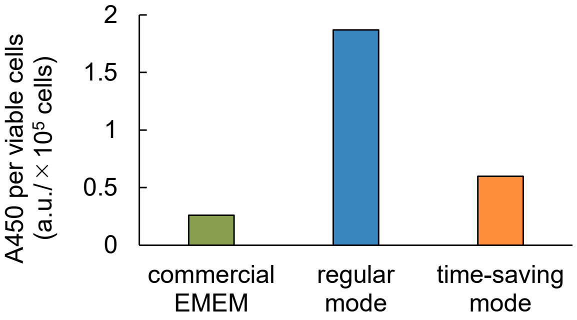

Supplementary Figure 10 Cell culture of CHO-K1 in the media optimized with HeLa. The cellular A450 values at 168 h in the optimized media predicted by the regular and time-saving modes are compared to that in EMEM. The mean A450 (N=3) ratio to mean cell concentration (N=3) is shown. Note that 0.5 mM of proline was additionally supplied because CHO-K1 has auxotrophic for proline. An arbitrary unit is shown as a.u.
